# The Na^+^/H^+^ Exchanger 3 in the Intestines and the Proximal Tubule of the Kidney: Localization, Physiological Function, and Key Roles in Angiotensin II-Induced Hypertension

**DOI:** 10.3389/fphys.2022.861659

**Published:** 2022-04-19

**Authors:** Sarah M. Nwia, Xiao Chun Li, Ana Paula de Oliveira Leite, Rumana Hassan, Jia Long Zhuo

**Affiliations:** ^1^Tulane Hypertension and Renal Center of Excellence, Tulane University School of Medicine, New Orleans, LA, United States; ^2^Department of Physiology, Tulane University School of Medicine, New Orleans, LA, United States

**Keywords:** angiotensin II, Na^+^/H^+^ exchanger 3, hypertension, kidney, proximal tubule

## Abstract

The sodium (Na^+^)/hydrogen (H^+^) exchanger 3 (NHE3) is one of the most important Na^+^/H^+^ antiporters in the small intestines of the gastrointestinal tract and the proximal tubules of the kidney. The roles of NHE3 in the regulation of intracellular pH and acid–base balance have been well established in cellular physiology using *in vitro* techniques. Localized primarily on the apical membranes in small intestines and proximal tubules, the key action of NHE3 is to facilitate the entry of luminal Na^+^ and the extrusion of intracellular H^+^ from intestinal and proximal tubule tubular epithelial cells. NHE3 is, directly and indirectly, responsible for absorbing the majority of ingested Na^+^ from small and large intestines and reabsorbing >50% of filtered Na^+^ in the proximal tubules of the kidney. However, the roles of NHE3 in the regulation of proximal tubular Na^+^ transport in the integrative physiological settings and its contributions to the basal blood pressure regulation and angiotensin II (Ang II)-induced hypertension have not been well studied previously due to the lack of suitable animal models. Recently, novel genetically modified mouse models with whole-body, kidney-specific, or proximal tubule-specific deletion of NHE3 have been generated by us and others to determine the critical roles and underlying mechanisms of NHE3 in maintaining basal body salt and fluid balance, blood pressure homeostasis, and the development of Ang II-induced hypertension at the whole-body, kidney, or proximal tubule levels. The objective of this invited article is to review, update, and discuss recent findings on the critical roles of intestinal and proximal tubule NHE3 in maintaining basal blood pressure homeostasis and their potential therapeutic implications in the development of angiotensin II (Ang II)-dependent hypertension.

## Introduction

High blood pressure or hypertension has recently become a national epidemic of proportion in the public health. According to American Heart Association and American College of Cardiology’s recent estimates, 46% of American adults suffer from hypertension, which is defined as a systolic and diastolic blood pressure ≥130/80 mmHg (Whelton and Carey, 2017; Whelton et al., 2018). Important risk factors that increase an individual’s chances of developing hypertension include genetic predisposition, unhealthy diet, lack of physical activities or excess salt intake (Intersalt, 1988; Crowley et al., 2005; Coffman, 2011; Whelton and Carey, 2017; Whelton et al., 2018; Carey, 2019; Mattson, 2019; Harrison et al., 2021; Zhuo et al., 2021). A variety of treatments are available to treat hypertension, but nonpharmacological approaches are usually recommended first. Along with recommendations to lose weight, patients are also counseled to reduce sodium intake, increase potassium intake, and reduce alcohol consumption (Svetkey et al., 1999; Sacks et al., 2001; Siervo et al., 2015; Whelton et al., 2018). The average American sodium intake for those 1 year and older totals 3,440 mg/day, almost 1,500 mg/day higher than the recommended limit of 2,300 mg/day (Svetkey et al., 1999; Sacks et al., 2001; Siervo et al., 2015; DeSalvo, 2016). The effect of high dietary sodium intake on blood pressure has been well-studied and the causative relationship between increased dietary salt and increased blood pressure has been well-established by salt-sensitive hypertension animal models (Rapp, 1982; Intersalt, 1988; Joe and Shapiro, 2012; DeSalvo, 2016; Zhuo and Li, 2018; Mattson, 2019; Filippini et al., 2021; Harrison et al., 2021). Dietary sodium is absorbed in the gastrointestinal tract primarily by the Na^+^/H^+^ exchanger 3 (NHE3) and when the blood is filtered by the kidneys, NHE3 in the proximal tubule is chiefly responsible for >50% of sodium reabsorption, highlighting its important role in the regulation of physiological sodium and fluid balance, blood pressure homeostasis, and the pathophysiology of hypertension (Schultheis et al., 1998a,b; Wang et al., 1999; Zhuo and Li, 2013; Li et al., 2015a,b).

If non-pharmacological approaches fail to control blood pressure, there are several classes of pharmacological agents available to treat hypertension. These antihypertensive therapeutics include diuretics targeting distal nephron Na^+^ transport, angiotensin-converting enzyme (ACE) inhibitors to inhibit the formation of a vasoactive peptide angiotensin II (Ang II), angiotensin II type 1 receptor blockers (ARBs), beta-blockers, and calcium channel blockers (Calhoun et al., 2008a; Whelton et al., 2018; Carey, 2019). For most hypertensive patients, treatment with a diuretic, ACE inhibitor, or ARB alone, or with a number of different classes of antihypertensive drugs will be able to control hypertension (Calhoun et al., 2008a,b; Muntner et al., 2017; Whelton et al., 2018; Carey, 2019). If a patient still has uncontrolled blood pressure while taking 3 different classes of medications or they require 4 or more different medications to control their blood pressure, they are considered to have resistant hypertension, or apparent treatment resistant hypertension (Calhoun et al., 2008b; Carey, 2013, 2016, 2019; Whelton et al., 2018). About 13% of United States adults have been diagnosed with resistant hypertension (Calhoun et al., 2008a,b; Carey, 2013, 2019; Whelton et al., 2018). This data indicates that the mechanisms of hypertension have yet to be completely understood and patients with resistant hypertension may benefit greatly from new knowledges and the development of novel hypertension treatments.

Against this background, we and others have recently hypothesized that the Na^+^/H^+^ exchanger 3 (NHE3) in small intestines and the proximal tubule of the kidney plays a key role in maintaining physiological blood pressure homeostasis and the development of Ang II-induced hypertension, and may serve a potential new therapeutic target in treating hypertension. To test our hypothesis, we and other laboratories have used novel mutant mouse models with whole body- (Schultheis et al., 1998a,b; Lorenz et al., 1999; Wang et al., 1999; Wang et al., 2001; Li et al., 2015b), kidney- (Woo et al., 2003; Noonan et al., 2005; Li et al., 2015a, 2019b; Fenton et al., 2017; Zhuo et al., 2021), or proximal tubule-specific deletion of NHE3 to study the important roles and underlying mechanisms of NHE3 in maintaining basal blood pressure homeostasis and the development of Ang II-induced hypertension (Li et al., 2018, 2019b; Zhuo et al., 2021). The objective of this invited article is to review, update, and discuss the localization, physiological function, and the roles of intestinal and proximal tubule NHE3 in maintaining basal blood pressure homeostasis and the development of Ang II-dependent hypertension.

## Overviews of the Na^+^/H^+^ Exchanger Family and Their Roles in Maintaining Normal Cellular Volume and Acid–Base Homeostasis

**Figure 1:**
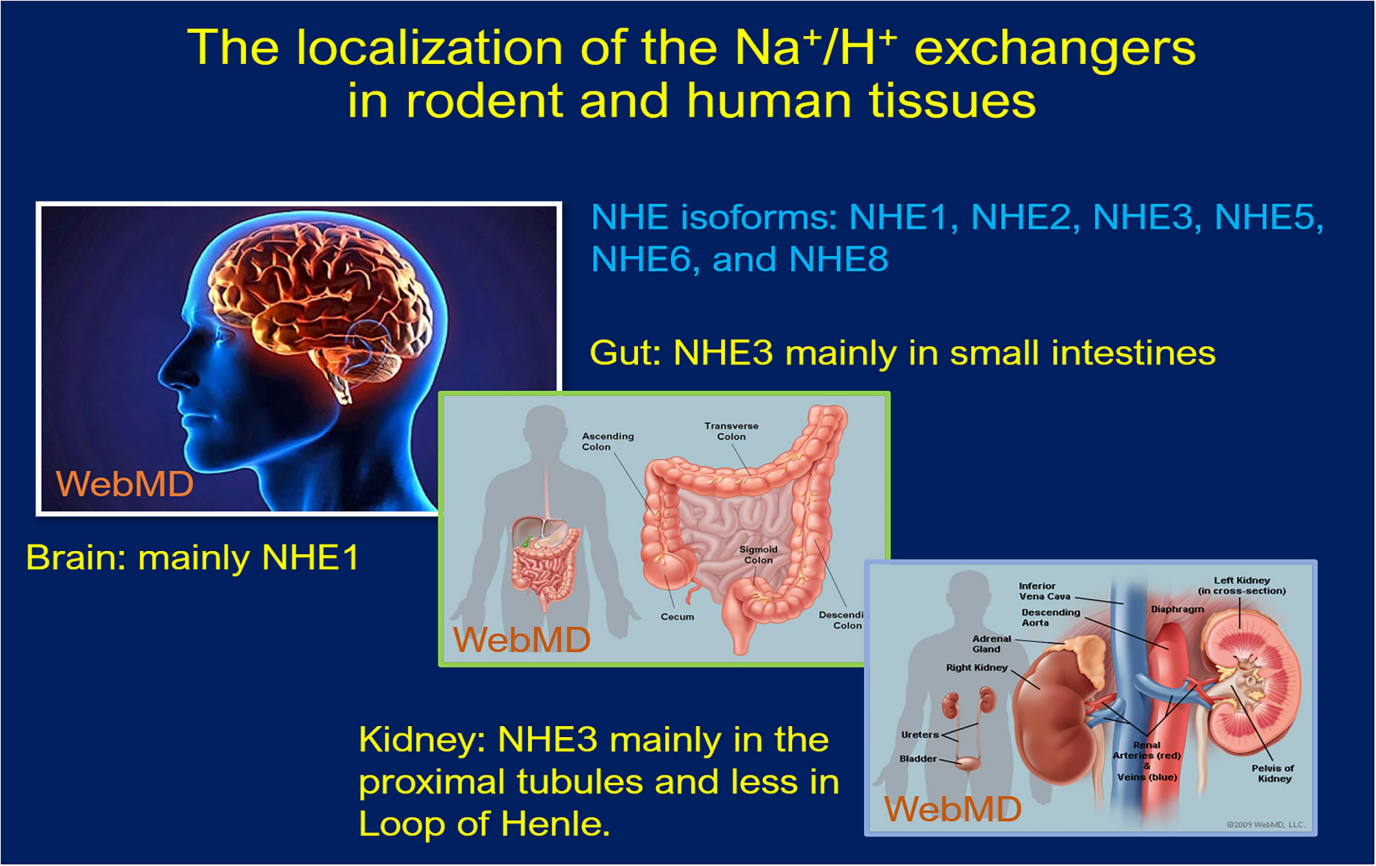
Schematic localization of the Na^+^/H^+^ exchanger expression in human and rodent tissues. NHE1 and NHE2 are expressed in almost every tissue type, including intestinal, kidney, testicular, gastric parietal, and skeletal muscle cells etc. NHE4 expression occurs in the GI tract, whereas NHE5 is most prominently expressed in the brain on glioma cells. NHE6–9 are specific in their intracellular localization found in endosomes, while NHE7 is localized to the trans-Golgi network along with early and recycling endosomes. NHE8 is also an intracellular NHE protein, especially in late endosomes and facilitates alkalinization of the organelle.

The sodium (Na^+^) and proton (H^+^) antiport cross the cell membranes plays a fundamental role in maintaining normal cellular volume and acid–base homeostasis (Yun et al., 1995; Donowitz et al., 2013; Pedersen and Counillon, 2019). The presence of a direct coupling between Na^+^ and H^+^ flux across the brush border membranes of rat small intestines and the kidney was demonstrated in 1970s (Murer et al., 1976). The genes encoding a Na^+^/H^+^ exchanger was subsequently cloned in humans (Sardet et al., 1989; Takaichi et al., 1992), rabbits (Tse et al., 1992, 1993; Collins et al., 1993), and rats (Orlowski et al., 1992; Wang et al., 1993). The Na^+^/H^+^ exchanger family is now known to have nine distinct isoforms (NHE1-NHE9), based on the order of their molecular cloning, each serving a different role. These NHE isoforms range in size from 645 to 898 amino acids long, inserting into the plasma membrane with the N-terminal facing the extracellular space and the C terminal within the cytoplasmic domain (Sardet et al., 1989; Orlowski et al., 1992; Takaichi et al., 1992; Tse et al., 1992, 1993; Collins et al., 1993). The N-terminal is responsible for the exchange of solutes, while the cytosolic C-terminal mediates the hormonal regulation of the exchangers (Wakabayashi et al., 1992; Counillon et al., 1994; Yun et al., 1995; Pedersen and Counillon, 2019). Each isoform varies in its distinct tissue distribution, subcellular location, and level of expression in different tissues under different regulations (Figure 1; Counillon et al., 1994; Yun et al., 1995; Pedersen and Counillon, 2019).

NHE1 is expressed in almost every tissue type, responsible for maintaining appropriate intracellular volume and cytosolic pH (Biemesderfer et al., 1992; Yun et al., 1995; Evans et al., 1999; Pedersen and Counillon, 2019). NHE2 has been localized to intestinal, kidney, testicular, gastric parietal, and skeletal muscle cells, particularly on the plasma membranes (Bookstein et al., 1997; Chambrey et al., 1998; Schultheis et al., 1998b; Malakooti et al., 1999; Bachmann et al., 2004). NHE4 can be found along the GI tract, as well as the basolateral membranes of the thick ascending limb and distal convoluted tubule of the nephron, where it plays an important role in maintaining cellular pH homeostasis through aldosterone signaling (Chambrey et al., 2001; Bourgeois et al., 2010; Arena et al., 2012; Pedersen and Counillon, 2019). NHE5 is most prominently expressed in the brain on glioma cells and plays a role in the regulation of growth factor and integrin signaling (Attaphitaya et al., 1999; Kurata et al., 2019). NHE6-9 are specific in their intracellular localization. NHE6 and 9 are found in endosomes, while NHE7 has been localized to the trans-Golgi network along with early and recycling endosomes (Xinhan et al., 2011; Pedersen and Counillon, 2019; Kucharava et al., 2020). NHE8 is also an intracellular NHE protein but is a component of late endosomes and facilitates alkalinization of the organelle (Goyal et al., 2005; Lawrence et al., 2010; Pedersen and Counillon, 2019).

The roles of NHE1, NHE2, and NHE8 along the GI tract or the kidney remain poorly understood, but it is most likely that their respective contributions to overall salt and fluid, acid and base balance, Na^+^ absorption in the gastrointestinal tract, and Na^+^ reabsorption in the kidney may be very limited (Yun et al., 1995; Zachos et al., 2005; Bourgeois et al., 2010; Pedersen and Counillon, 2019). Indeed, the role of NHE1 was investigated through a whole-body genetic deletion model (*Nhe1^−/−^*), but the results yielded CNS pathologies instead such as ataxia and seizures and no evidence of abnormal Na^+^ reabsorption or blood pressure phenotypes were noted (Cox et al., 1997). NHE2 has also been investigated as a potential contributor to sodium and water reabsorption along the GI tract; however, a *Nhe2^−/−^* knockout model demonstrated no differences in GI tract reabsorption when compared to the wild-type (WT), with similar results emerging from a pharmacological inhibition study (Schultheis et al., 1998b; Ledoussal et al., 2001a,b; Gawenis et al., 2002; Bachmann et al., 2004; Bailey et al., 2004; Guan et al., 2006; Engevik et al., 2013). Like *Nhe1*^*−*/−^ and *Nhe2^−/−^* models, *Nhe8^−/−^* mouse models also demonstrated no significant difference in serum Na^+^ levels and exhibited a lack of diarrhea phenotype indicating the limited role of NHE8 in intestinal Na^+^ absorption and fluid balance (Xu et al., 2012; Xu et al., 2013). Thus, there is clear lack of evidence supporting any important role of these NHE isoforms in mediating or regulating sodium absorption and maintaining body salt and fluid balance or blood pressure homeostasis. Because there is no direct evidence in the literature for these above-mentioned NHEs to play a significant role in regulating intestinal and kidney Na^+^ transport and maintaining basal body salt and fluid balance and blood pressure homeostasis, this article will only focus on NHE3 in the gastrointestinal tract and the kidney.

## Localization of NHE3 and Molecular Mechanisms Regulating NHE3 Expression in Gastrointestinal and Kidney Tissues

NHE3 is an antiporter that is expressed mainly on the apical membrane but can be sequestered intracellularly within endosomes depending on body salt and fluid, acid and base balance, blood pressure homeostasis, or hemodynamic factors (Wu et al., 1996; Chow, 1999; Donowitz et al., 2013; Pedersen and Counillon, 2019; Zhuo et al., 2021). NHE3 has been localized primarily in abundance to the small intestines of the gastrointestinal tract and the proximal tubules and the thick ascending limbs of the kidney (Biemesderfer et al., 1993; Kulaksiz et al., 2001; He and Yun, 2010; Shawki et al., 2016; Pedersen and Counillon, 2019). Large intestines also express NHE3 but with a much lower level than small intestines (Bookstein et al., 1997; Gawenis et al., 2002; Bradford et al., 2009; He and Yun, 2010; Pedersen and Counillon, 2019). Indeed, the levels of NHE3 expression in small and large intestines are corresponding to their respective contributions to absorbing ingested sodium loads. In the kidney, NHE3 is expressed primarily along the apical membranes of the proximal tubules, especially in early S1 and S2 segments, and its expression gradually decreases throughout the thick ascending limb of the loop of Henle (Biemesderfer et al., 1993; Wu et al., 1996; Cox et al., 1997; Chow, 1999; Brooks et al., 2001; Wade et al., 2001; Bacic et al., 2003; Zhuo et al., 2021).

The expression of NHE3 in the gastrointestinal tract and the kidney may be regulated by diverse molecular mechanisms and hormonal factors (Wu et al., 1996; D’Souza et al., 1998; Hu et al., 2001; Leong et al., 2002; Fuster et al., 2007; Li et al., 2009a, 2012; Kemp et al., 2014, 2016; Castelo-Branco et al., 2017). A number of mechanisms have been shown to regulate the level of NHE3 expression, but the majority of these regulators can be divided into four groups—phosphorylation, trafficking, protein–protein interaction, and transcriptional regulation (Zhao et al., 1999, 2004; He and Yun, 2010; Pedersen and Counillon, 2019). NHE3 can be phosphorylated by a variety of protein kinases including protein kinase A, which downregulates signal transduction pathways, and casein kinase 2, which upregulates NHE3 trafficking to the plasma membranes (Kurashima et al., 1997; Zhao et al., 1999; Kocinsky et al., 2005; Sarker et al., 2008). NHE3 trafficking describes the ability of NHE3 to be recycled between intracellular compartments and the plasma membrane *via* a clathrin-mediated pathway (D'Souza et al., 1998). However, there is accumulating evidence that under physiological conditions, NHE3 is equally distributed between the body (active) and base (less active) of the microvilli in brush border membranes (Biemesderfer et al., 2001). In response to the changes in renal perfusion pressure, hormonal, or pharmacological factors, NHE3 may be translocated between the body and base of the microvilli (Yip et al., 1998; Yang et al., 2007; Riquier-Brison et al., 2010). Indeed, acute hypertension or acute ACE inhibition induced NHE3 translocation from the body to the base of microvilli in the rat kidney (Yip et al., 1998; Yang et al., 2007). The vasoactive peptide hormone angiotensin II (Ang II) appears to regulate membrane NHE3 abundance in a biphasic manner (Leong et al., 2002; Yang et al., 2007; Li et al., 2009b, 2012; Riquier-Brison et al., 2010; Li and Zhuo, 2011). A physiological concentration of Ang II increases apical membrane NHE3 protein abundance, while a high pressor concentration of Ang II increases NHE3 translocation from the membranes and reduces the number of transporters on the membranes (Leong et al., 2002; He et al., 2010; Riquier-Brison et al., 2010; Li and Zhuo, 2011).

Another mechanism by which the expression of NHE3 on the plasma membrane can be regulated is *via* protein–protein interactions. One example of this in the proximal tubules is the Na^+^/H^+^ exchanger regulatory factor (NHERF1), which is activated by dopamine and results in the inactivation of the NHE3 transporter through the formation of a multiprotein signaling complex (Wade et al., 2001; Weinman et al., 2001; He et al., 2010; Giral et al., 2012). Finally, the regulation of NHE3 expression may involve transcriptional regulation, which is an insidious process when compared to the other mechanisms (He and Yun, 2010; Pedersen and Counillon, 2019). In addition to their effect on exocytosis, insulin, dexamethasone and glucocorticoids activate (Bobulescu et al., 2005; Fuster et al., 2007; Wang et al., 2007); whereas dopamine, cAMP, and cGMP inhibit the exocytosis or transcription levels of NHE3 (Yun et al., 1997; Zizak et al., 1999; Hu et al., 2001; Bacic et al., 2003; Cha et al., 2005). Acute treatment with parathyroid hormone (PTH) appeared to inhibit NHE3 translocation from the base of microvilli (Yang et al., 2004), whereas long-term treatment with PTH decreased NHE3 mRNA levels due to a PKA-dependent inhibitory effect on the NHE3 promoter (Bezerra et al., 2008). Taken together, the expression of NHE3 on the plasma membrane is regulated by a complex system, allowing it to adapt to body salt and fluid balance, vasoactive hormones, and molecular and cellular stressors to efficiently regulate cellular acid and base, maintain body salt and fluid balance and blood pressure homeostasis.

## Physiological Function of Gastrointestinal NHE3 in the Regulation of Body Salt and Fluid Balance and Blood Pressure Homeostasis

**Figure 2:**
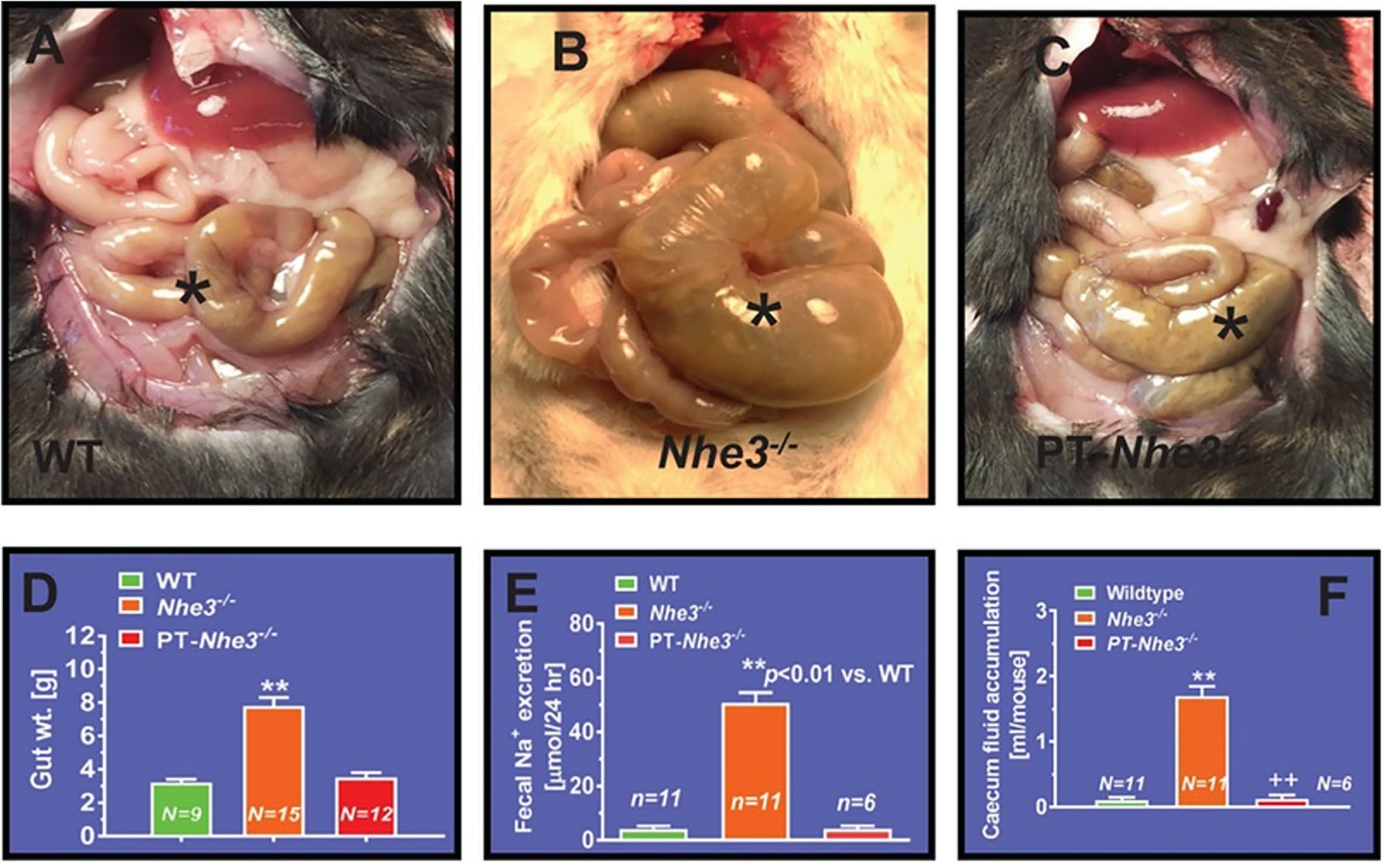
Proximal tubule-specific deletion of NHE3 does not significantly alter the intestinal structural and absorptive phenotypes in PT-*Nhe3^−/−^* mice. **(A)** A representative normal caecum segment between small and large intestines in a WT mouse (^*^). **(B)** A representative cecum segment between small and large intestines in a global *Nhe3^−/−^* mouse, showing the extremely enlarged cecum segment accumulated with a large volume of fluid content inside (^*^). **(C)** A representative cecum segment between small and large intestines in a PT-*Nhe3^−/−^* mouse, showing the lack of enlarged cecum segment and no accumulation of a large volume of fluid content in the cecum segment (^*^). **(D)** The overall gut weight more than doubled in global *Nhe3^−/−^* mice than WT and PT-*Nhe3^−/−^* mice (*p* < 0.01). **(E)** Twenty-four hours fecal Na^+^ excretion was ~10-time higher in global *Nhe3^−/−^* mice than WT and PT-*Nhe3^−/−^* mice (*p* < 0.01). **(F)** Accumulation of fluid content in the cecum segment was ~10 times higher in global *Nhe3^−/−^* mice than WT and PT-*Nhe3^−/−^* mice (*p* < 0.01). There were no differences in the overall gut weight and 24 h fecal Na^+^ excretion between WT and PT-*Nhe3^−/−^* mice. Reproduced from Li et al., 2018 with permission from the copyright holder.

**Figure 3:**
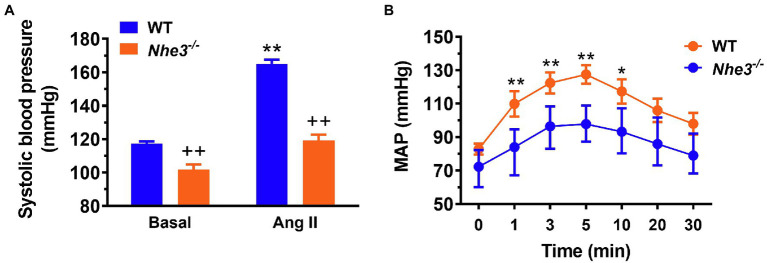
Angiotensin II-induced hypertension is attenuated in global *Nhe3^−/−^* mice with transgenic rescue of the NHE3 gene in small intestines using the small intestine-specific IFABP promoter, tg*Nhe3*^−/−^ **(A,B)**. Note that basal blood pressure remains significantly lower in tg*Nhe3*^−/−^ mice than WT mice **(A)**. ^*^p < 0.05, and ^**^p < 0.01 vs. their basal level; ^++^*p* < 0.01 vs. WT mice. Reproduced from Li et al. (2019a) with permission from the copyright holder.

In contrast to other NHE isoforms, NHE3 is the major isoform in the NHE family and plays the most significant role in regulating the absorption of sodium within the gastrointestinal tract (Schultheis et al., 1998a; Gawenis et al., 2002; Woo et al., 2003; Bradford et al., 2009; Li et al., 2015a,b; Shawki et al., 2016; Zhuo et al., 2021). This is strongly supported by integrative studies with generation of global or whole-body NHE3 deletion mouse models (*Nhe3^−/−^*) to investigate its role in the intestinal absorption of sodium. Schultheis et al. (1998a) were the first to generate and reported the gastrointestinal phenotypes of the *Nhe3^−/−^* mouse model. Although these *Nhe3^−/−^* mice were able to survive, they developed severe diarrhea and Na^+^ wasting phenotypes, with a large increase in 24-h fecal Na^+^ excretion when compared to the WT or proximal tubule-specific *Nhe3^−/−^* mice, PT- *Nhe3^−/−^* (Figure 2; Schultheis et al., 1998a; Li et al., 2015b, 2018b). Additionally, there was a major upregulation in epithelial Na^+^ channel (ENaC) and H^+^/K^+^ ATPase activity, but with near-normal blood gas, pH, and electrolyte balance, indicating that a compensatory mechanism might be at work (Schultheis et al., 1998a). These results indicated that NHE3 is the primary transporter responsible for the absorption of dietary sodium in the intestines and therefore contributes considerably to the maintenance of sodium and fluid balance. Despite its key role in the absorption of dietary sodium, however, deletion of NHE3 along the gastrointestinal tract had no significant impacts on the natriuresis response in *Nhe3^−/−^* mice (Li et al., 2015a,b). This is because the kidney acts to compensate for the loss of NHE3 in the proximal tubules and the loop of Henle by upregulating the expression of the renin–angiotensin–aldosterone system and other Na^+^ cotransporters, such as sodium and glucose cotransporter 2 (SGLT2), Na^+^/K^+^-ATPase, sodium bicarbonate cotransporter, and aquaporin 1 (AQP1; Li et al., 2015a,b). Nevertheless, basal systolic, diastolic, and mean arterial blood pressure are consistently about 15 to 20 mmHg lower in adult male and female global *Nhe3^−/−^* mice than WT mice (Figure 3), therefore affirming an important role of intestinal and kidney NHE3 in maintaining physiological blood pressure homeostasis (Schultheis et al., 1998a; Li et al., 2015b, 2018b).

**Figure 4:**
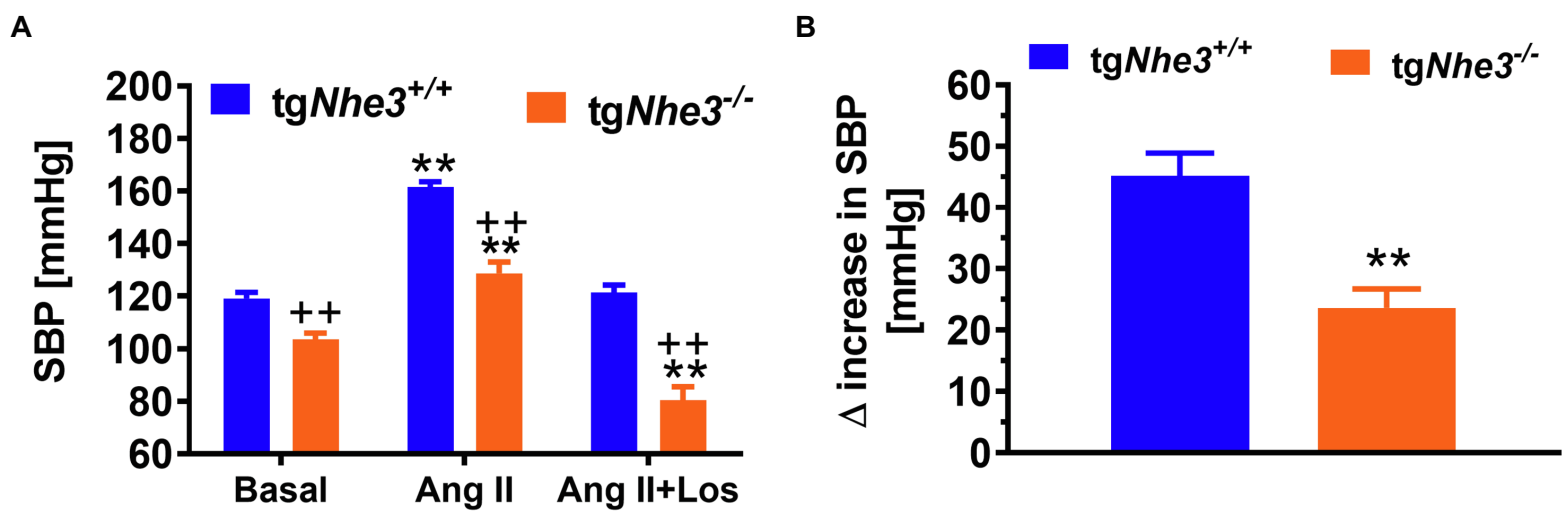
Angiotensin II-induced hypertension is attenuated in global *Nhe3^−/−^* mice with transgenic rescue of the NHE3 gene in small intestines using the small intestine-specific IFABP promoter, tg*Nhe3^−/−^* **(A,B)**. Note that basal blood pressure remains significantly lower in tg*Nhe3^−/−^* mice than WT mice **(A)**. ^**^p < 0.01 vs. their basal level; ^++^*p* < 0.01 vs. WT mice. Reproduced from Li et al. (2019a) with permission from the copyright holder.

To further differentiate the roles of intestinal and kidney NHE3 in the regulation of Na^+^ absorption and reabsorption and blood pressure, Woo et al. (2003) and Li et al. (2015a) have generated and studied a different *Nhe3^−/−^* mouse model, tg*Nhe3^−/−^*, with transgenic rescue of the NHE3 gene selectively in small intestines using the small intestine-specific IFABP promoter (Woo et al., 2003; Noonan et al., 2005; Li et al., 2015a). In this mouse model, overall salt wasting phenotypes including severe diarrhea, marked increased fecal Na^+^ excretion, and lowered basal blood pressure were moderately but significantly improved in tg*Nhe3^−/−^* mice (Woo et al., 2003; Noonan et al., 2005; Li et al., 2015a), but all these abnormal phenotypes persisted in tg*Nhe3^−/−^* mice (Li et al., 2015a; Zhuo et al., 2021). Indeed, diarrhea persisted, and basal blood pressure remained significantly lower in tg*Nhe3^−/−^* mice (Figure 4; Li et al., 2015a; Zhuo et al., 2021). Recently, Xue et al. (2020, 2022) have generated a new inducible intestinal epithelial cell-specific NHE3 knockout mouse model, which showed similar intestinal abnormal absorptive phenotypes, mimicking congenital sodium diarrhea with enhanced phosphate. Thus, the development of moderate to severe diarrhea due to intestinal Na^+^ absorptive defect is a major phenotype in global and intestine-specific *Nhe3^−/−^* mice (Schultheis et al., 1998a; Li et al., 2015a,b; Xue et al., 2020, 2022).

Another interesting and consistent finding in studying global *Nhe3^−/−^* and tg*Nhe3^−/−^* mice is that these mice develop striking structural abnormalities in small intestines (Schultheis et al., 1998a,b; Li et al., 2015a,b). The structural abnormalities show that all intestinal segments were significantly enlarged in *Nhe3^−/−^* mice, with extremely enlarged cecum segment containing a large amount of accumulated fluid (Schultheis et al., 1998a; Li et al., 2015a,b). These abnormalities were also found in *Nhe3^−/−^* mice with transgenic rescue of the NHE3 gene selectively in small intestines (Li et al., 2015a). Since mice with deletion of NHE3 selectively in the proximal tubules of the kidney do not develop these intestinal structural abnormalities, and severe diarrhea and Na^+^ wasting phenotypes (see below; Li et al., 2018, 2019a), our studies suggest that NHE3 in the gastrointestinal tract may likely play an important role in the development of the gut in addition to its major role in mediating Na^+^ absorption. However, a recently developed, inducible intestine-specific *Nhe3^−/−^* mouse model does not show any intestinal structural abnormalities as *Nhe3^−/−^* or tg*Nhe3^−/−^* mice do (Xue et al., 2020, 2022). The reasons underlying these differences between global *Nhe3^−/−^*, transgenic tg*Nhe3^−/−^*, and intestine-specific *Nhe3^−/−^* mice remain unknown. One likely explanation may be that the inducible intestine-specific *Nhe3^−/−^* model is a short-term deletion model in adult mice lasting only for a few weeks, which may not be long enough to reveal any structural development problems. Further studies are necessary to uncover the molecular mechanisms underlying the development of structural abnormalities in small intestines in *Nhe3^−/−^* and tg*Nhe3^−/−^* mice.

## Physiological Function of NHE3 in the Kidney Proximal Tubules in the Regulation of Body Salt and Fluid Balance and Blood Pressure Homeostasis

It is well recognized that among all Na^+^ transporters and cotransporters, NHE3 is the most critical Na^+^ transporter responsible for Na^+^ reabsorption within the kidney (Lorenz et al., 1999; Wang et al., 1999; Zhuo and Li, 2013; Li et al., 2018, 2019a; Zhuo et al., 2021). Specifically, the proximal tubules are responsible for reabsorbing 65%–70% of glomerular filtered Na^+^ and water loads, whereas NHE3 in the proximal tubules is directly and indirectly responsible for >50% of glomerular filtered Na^+^ reabsorption (Schultheis et al., 1998a; Lorenz et al., 1999; Wang et al., 1999; Li et al., 2015a,b; Zhuo et al., 2021). To further investigate the physiological function of NHE3 in the kidney, pan-renal tubule-specific and proximal tubule-specific *Nhe3^−/−^* mouse models have been generated and studied, respectively, during recent years. A whole-kidney tubule epithelia-specific *Nhe3^−/−^* mouse model was generated by cross breeding NHE3-floxed mice with *Pax8-Cre* mice (Fenton et al., 2015, 2017). This scientific approach is based on the premise that Pax8 is only expressed along the renal tubules, thus in theory, NHE3 is expected to be deleted from all nephron segments in the kidney, including proximal tubule segments (S1–S3) and the ascending limb of the loop of Henle in this *Pax8-Cre/NHE3-*floxed mouse model. These mice were found to have fully compensated plasma ion concentrations, osmolality, and pH when compared to the WT counterpart. These findings of normal blood biochemical phenotypes in *Pax8-Cre/NHE3-*floxed mice have effectively alleviated a major concern that genetic deletion or therapeutically targeting of NHE3 in the kidney may cause body acid-base, and salt and fluid imbalance. Alternatively, these results strongly suggest that these mice are able to adequately compensate for the loss of NHE3 function in the kidney to maintain normal body salt and fluid, acid and base balance (Fenton et al., 2015, 2017). However, despite these similar blood biochemistries, these mice increased fluid intake and urine flow rate, with decreased urine osmolality and increased urine pH (Fenton et al., 2015, 2017). Intra-arterial blood pressure was found to be significantly lower in *Pax8-Cre/NHE3-*floxed mice likely due to increased natriuretic responses. Furthermore, these mice demonstrated an increased sensitivity to dietary NaCl, along with a 20% decrease in GFR (Fenton et al., 2017). Taken together, this study using *Pax8-Cre/NHE3-*floxed mice provides important evidence for an important role of NHE3 along the entire nephron segments contributing to body salt and fluid balance and blood pressure homeostasis.

**Figure 5:**
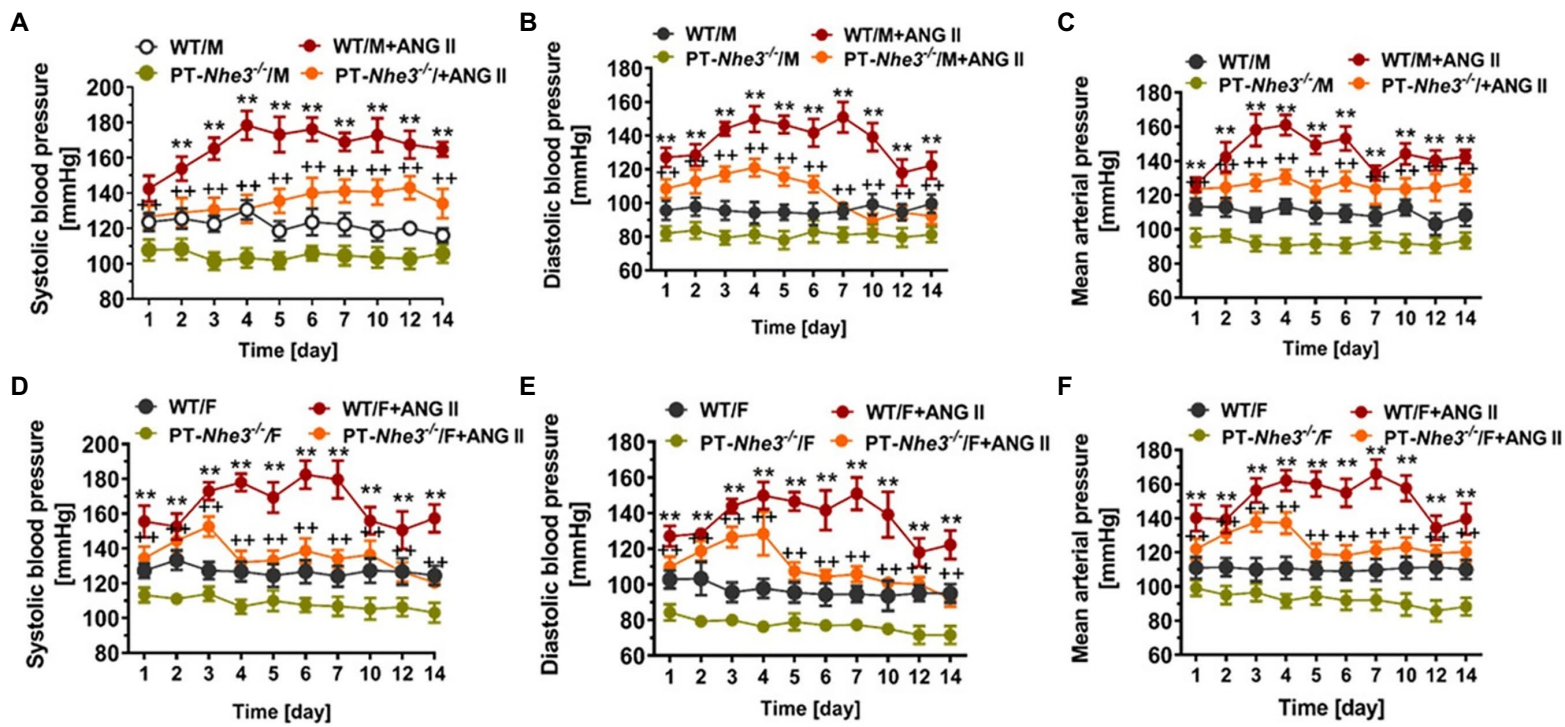
Systolic, diastolic and mean arterial blood pressure responses to a high pressor dose of ANG II infusion, 1.5 mg/kg/day, i.p., in conscious adult male **(A-C)** and female **(D-F)** WT and PT-*Nhe3^−/−^* mice, as measured continuously for 14 days using the direct implanted telemetry technique. Note that basal blood pressure levels were about 12–15 mmHg lower in PT-*Nhe3^−/−^* than WT mice, and that Ang II-induced hypertension was attenuated in male **(A-C)** and female **(D-F)** PT-*Nhe3^−/−^* mice. ^**^p < 0.01 vs. WT time-control group; ^++^*p* < 0.01 vs. PT-*Nhe3^−/−^* time-control group, respectively. *N* = 5–12 per group. Reproduced from Li et al. (2019b) with permission.

However, *Pax8-Cre/NHE3-*floxed mice is at best a whole kidney tubular epithelia-specific *Nhe3^−/−^* mouse model, the contributions of NHE3 in the proximal tubules and the loop of Henle are difficult to be separated in this mouse model. In previous micropuncture studies, Vallon et al. (2000); Gekle et al. (2004) showed that luminal perfusion of the rat proximal tubule and loop of Henle with a NHE3 inhibitor S3226 inhibited proximal tubular reabsorption by ~30% without altering fluid and Na^+^ reabsorption in the Loop of Henle. This study is largely consistent with the micropuncture findings that proximal tubular fluid or bicarbonate reabsorption was decreased by about 50% in global *Nhe3^−/−^* mice (Lorenz et al., 1999; Wang et al., 1999). Since the proximal tubule is responsible for 65%–70% of Na^+^ reabsorption in the kidney, whereas NHE3 in the proximal tubule is directly and indirectly responsible for over 50% of Na^+^ reabsorption in the kidney, our group recently generated and studied a new *Nhe3^−/−^* mouse model with proximal-tubule specific deletion of NHE3 using the *SGLT2-Cre/NHE3*-floxed approach (PT-*Nhe3^−/−^*; Li et al., 2018, 2019b; Zhuo et al., 2021). This new mutant mouse model allowed us to directly test the hypothesis that NHE3 in the proximal tubule is primarily responsible for maintaining body salt and fluid balance and blood pressure homeostasis in part by modulating the pressure-natriuresis response (Li et al., 2018, 2019b; Zhuo et al., 2021). In contrast to the whole-body *Nhe3^−/−^* (Schultheis et al., 1998a; Li et al., 2015a,b) or the tg*Nhe3^−/−^* mouse model (Woo et al., 2003; Noonan et al., 2005; Li et al., 2015a), this proximal tubule-specific *Nhe3^−/−^* mouse model did not show the abnormalities in both structures and Na^+^ absorption in the gastrointestinal tract (Li et al., 2018, 2019b, 2021). No diarrhea phenotype was developed in this proximal tubule-specific *Nhe3^−/−^* mouse model since no difference was found in 24 h fecal Na^+^ excretion between WT and PT-*Nhe3^−/−^* mice (Figure 2). Additionally, there were no differences in plasma pH, plasma ions or bicarbonate, and hematocrit between WT and PT-*Nhe3^−/−^* mice, suggesting that loss of NHE3 function in the proximal tubules does not cause abnormal body acid and base, as well as body electrolyte and fluid balance (Li et al., 2018, 2019b, 2021). However, 24-h urine (diuresis) and urinary Na^+^ (natriuresis) and K^+^ excretion were significantly increased under basal physiological conditions. These kidney phenotypes were associated significantly decreased basal systolic, diastolic, and mean arterial blood pressure (−12 to −15 mmHg) in both male and female PT-*Nhe3^−/−^* mice when measured *via* telemetry (Figure 5; Li et al., 2018, 2019b, 2021). When compared to the wild type, the pressure-natriuresis response was significantly augmented in PT-*Nhe3^−/−^* mice in response to similar increases in renal perfusion pressure. These findings strongly support the hypothesis that NHE3 in the proximal tubule of the kidney is responsible for a significant bulk of Na^+^ and water reabsorption, and that the presence or loss of NHE3 function in the proximal tubules alone is sufficient to alter basal blood pressure homeostasis.

## NHE3 in the Gastrointestinal and the Proximal Tubules of the Kidney Plays an Important Role in the Development of Ang II-Induced Hypertension

The renin–angiotensin–aldosterone system (RAAS) has been well established as an important vasoactive and humoral system in the regulation of blood pressure, with its major components widely expressed in different tissues (De Gasparo et al., 2000; Kobori et al., 2007; Navar et al., 2011; Forrester et al., 2018). However, the kidney RAAS plays a dominant role (Harris and Navar, 1985; Crowley et al., 2005, 2006; Li et al., 2009b, 2011, 2018, 2021; Gurley et al., 2011; Yang and Xu, 2017; Kemp et al., 2022). The juxtaglomerular (JG) cells in the renal cortex express prorenin, which is cleaved into renin when the JG cells are activated in response to stimuli like decreased blood pressure, sympathetic activation, or decreased sodium delivery to the macula densa (Kobori et al., 2007; Chappell, 2012; Zhuo et al., 2013; Sequeira-Lopez and Gomez, 2021). Renin is released into the kidney as well as in the circulation to cleave the liver-derived substrate angiotensinogen (AGT) to produce angiotensin I (Ang I), which is the precursor for the active peptide Ang II (Kobori et al., 2007; Forrester et al., 2018). Ang I must then be converted into the biologically active Ang II by the angiotensin-converting enzyme (ACE; Kobori et al., 2007; Chappell, 2012; Zhuo et al., 2013; Sequeira-Lopez and Gomez, 2021). While other Ang peptides may possess different biological activities, Ang II is the key active peptide in the regulation of blood pressure *via* acting upon AT_1_ and AT_2_ receptors, which have counter-regulatory properties (Harris and Navar, 1985; Crowley et al., 2005, 2006; Li et al., 2009b, 2011, 2018, 2021; Gurley et al., 2011; Carey, 2017; Kemp et al., 2022).

In the proximal tubules of the kidney, Ang II activates G protein-coupled AT_1_ (AT_1a_) receptors on apical and basolateral membranes, which mediates G_q/11_/phospholipase C/IP_3_/protein kinase C signaling pathways and increases the expression of NHE3 and Na^+^ reabsorption in the proximal tubules (De Gasparo et al., 2000; Kobori et al., 2007; Li et al., 2009b; Li and Zhuo, 2011; Navar et al., 2011; Forrester et al., 2018; Li et al., 2018). Ang II may also indirectly activate the G_i_/cAMP/protein kinase A signaling pathways to inhibit NHE3 activity in proximal tubule cells, as forskolin significantly increased cAMP production in OK cells and Ang II attenuated these receptors (Thekkumkara et al., 1998). Systemically, activation of AT_1_ receptors by Ang II leads to vasoconstriction, increased salt and water retention, sympathetic stimulation, and increased aldosterone synthesis (De Gasparo et al., 2000; Kobori et al., 2007; Li et al., 2009a; Navar et al., 2011; Forrester et al., 2018). Conversely, activation of AT_2_ receptors by Ang II appears to provide counteract and protective effects against AT_1_ receptor-mediated effects by inducing vasodilation, diuresis and natriuresis (Siragy et al., 1999; Siragy and Carey, 1999; Kemp et al., 2014, 2016; Carey, 2017). Nevertheless, it is the AT_1_ receptor-mediated effects by Ang II play a predominant role in maintaining basal blood pressure homeostasis and the development of Ang II-dependent hypertension.

It is well-recognized that Ang II-induced or Ang II-dependent hypertension is caused by complex genetic, neural, and hormonal mechanisms involving AT_1_ receptor-mediated systemic, neural, adrenal, cardiovascular, gastrointestinal, and renal responses (Harris and Young, 1977; Harris and Navar, 1985; Navar et al., 1987; Crowley et al., 2005; Kobori et al., 2007; Forrester et al., 2018; Rucker et al., 2018; Li et al., 2019a). In both small intestines and the proximal tubules of the kidney, NHE3 is a recognized downstream target of Ang II-induced AT_1_ receptor activation, which increases NHE3 expression or activity and promotes Na^+^ absorption from small intestines and Na^+^ reabsorption from the proximal tubules (Levens et al., 1980, 1981; Saccomani et al., 1990 Jin et al., 1998; Li et al., 2009b, 2015a,b, 2019a; Zhuo et al., 2021). Increased Na^+^ absorption from the gut and reabsorption from the kidney without proportional increases in fecal and urinary Na^+^ excretion leads to body salt and fluid retention, one important mechanism underlying the development and progression of Ang II-dependent hypertension (Li et al., 2015a,b, 2019a). In a previous study, Gurley et al. (2011) showed that genetic deletion of AT_1a_ receptors in the renal proximal tubules protected against Ang II-induced hypertension in part by decreasing NHE3 expression in the kidney. However, whether and to what extent NHE3 in the gastrointestinal tract and the kidney directly contribute to the development of Ang II-induced hypertension have not been investigated previously.

Against this background, our group has tested this hypothesis by comparing systolic and mean arterial blood pressure responses to Ang II infusion in WT, whole-body *Nhe3^−/−^*, tg*Nhe3^−/−^* and PT-*Nhe3^−/−^* mice, respectively (Li et al., 2015a,b, 2018, 2019b, 2021). We tested two methods of Ang II infusion—an acute systemic infusion under anesthesia, and a chronic osmotic minipump infusion in conscious animals. Under both conditions, WT mice were found to have significantly increased systolic and mean arterial pressure, as expected. By contrast, whole-body *Nhe3^−/−^*, tg*Nhe3^−/−^* and PT-*Nhe3^−/−^* mice demonstrated significantly attenuated responses to acute or chronic Ang II infusions, with the decreases reaching about 50% of WT responses (Figures 3^5^; Li et al., 2015a,b, 2018, 2019b, 2021). Our results provide strong evidence for the 1st time an important role of NHE3 in the gastrointestinal tract and the proximal tubules of the kidney not only in maintaining basal blood pressure homeostasis but also in the development of Ang II-induced hypertension.

## NHE3 in the Proximal Tubules of the Kidney and the Development of Spontaneous Hypertension

The Na^+^/H^+^ exchanger 3 in the proximal tubules of the kidney has long been implicated in the development of hypertension in spontaneously hypertension in rats (SHRs; Thomas et al., 1988, 1990; Kelly et al., 1997; Aldred et al., 2000; LaPointe et al., 2002; Kemp et al., 2014, 2016, 2022). Whether NHE3 in the proximal tubules plays any role in hypertension in SHRs remains incompletely understood (Yip et al., 1998; Magyar et al., 2000; Panico et al., 2009; Crajoinas et al., 2014). Early *in vivo* micropuncture studies suggested that the development of hypertension in young SHRs up to 8 weeks of age involves sodium retention with significantly increased proximal tubule reabsorption (Jv; Thomas et al., 1988, 1990). Increased NHE3 expression and activity has been reported in the proximal tubules of young prehypertensive and adult hypertensive SHRs (Morduchowicz et al., 1989; Dagher and Sauterey, 1992; Hayashi et al., 1997; Kelly et al., 1997; Aldred et al., 2000; LaPointe et al., 2002; Crajoinas et al., 2010). These studies suggest that increased NHE3 expression and activity in the proximal tubules may contribute to the development of hypertension in SHRs by promoting proximal tubule Na^+^ reabsorption and inducing salt retention.

However, there is evidence that the expression and activity of NHE3 in the proximal tubules are decreased rather than increased in the development of hypertension in SHRs, and therefore NHE3 unlikely plays an important role in the development of spontaneous hypertension (Yip et al., 1998; Magyar et al., 2000; Panico et al., 2009; Crajoinas et al., 2014). In a micropuncture study, Panico et al. (2009) compared proximal tubule fluid reabsorption (Jv) between adult Wistar–Kyoto rats (WKY) and SHRs and found that Jv was in fact decreased by >50% in the proximal tubules of SHRs. The same study further showed that microperfusion of the proximal tubules with a highly potent NHE3 inhibitor, S-1611, decreased Jv in WKY rats but not in SHRs, suggesting that NHE3 stimulates proximal tubule Na^+^ reabsorption in WKY rather than in SHRs (Panico et al., 2009). In another studies, Crajoinas et al. (2010, 2014) microperfused proximal tubules to determine NHE3-mediated bicarbonate reabsorption between young prehypertensive SHR (5-wk-old) and adult SHR (14-wk-old), and age-matched WKY rats. NHE3 transport activity was found to increase significantly in the proximal tubules of pre-hypertensive SHRs, but not in adult SHRs, when compared with age matched WKY rats (Crajoinas et al., 2010). The lower NHE3 activity in adult SHRs may probably be due to higher phosphorylation of NHE3 at serine 552 (Crajoinas et al., 2010, 2014). More importantly, however, lower NHE3 activity and reduced proximal tubule Na^+^ reabsopriton in adult SHRs may be due to the redistribution of NHE3 proteins from the brush border (or apical membranes) to the base of the microvilli of proximal tubules (Zhang et al., 1996; Yip et al., 1998; Magyar et al., 2000). Using confocal immunofluorescent microscopic imaging, Yip et al. (1998) demonstrated that NHE3 was localized in the brush border of the microvilli in the proximal tubules of young prehypertensive SHRs, whereas in adult SHRs with established hypertension, it was found at the base of the microvilli of the proximal tubules. Alternatively, Magyar et al. (2000) used the subcellular fractionation of the renal cortex for subcellular localization of NHE3 proteins in the proximal tubules of young and adult SHRs, and compared with that of age matched Sprague–Dawley (SD) rats in response to the development of acute or chronic hypertension. In adult SD rats with acute hypertension, cortical apical NHE3 was found to redistribute from fractions 4–6 to fractions 5–7, even to fractions 8–10, likely corresponding to the base of the microvilli (Magyar et al., 2000). In adult SHRs with further acute increases in blood pressure, the redistribution peak of renal cortical NHE3 was similar to that of SD rats with acute hypertension. Similar subcellular redistribution of NHE3 was found in adult SHRs with chronic hypertension (Magyar et al., 2000). These studies suggest that NHE3 proteins in the proximal tubules redistribute constantly between the brush border and the base of the microvilli in response to the changes in blood pressure. However, how the increased or decreased NHE3 expression and activity correlates with the subcellular redistribution of NHE3 proteins in the proximal tubules before and after the development of hypertension in SHRs remains incompletely understood.

In addition to well-recognized AT_1_ receptor-mediated effects on NHE3 expression and activity, a defect in AT_2_ receptor-mediated inhibition of NHE3 expression and activity has been reported in the proximal tubules of SHRs, which may contribute to the development of hypertension in SHRs (Kemp et al., 2014, 2016, 2022; Zhuo and Li, 2019). Although Ang II also bind the AT_2_ receptors, the predominant agonist for this receptor may be the downstream metabolite of Ang II, i.e., Ang III (Padia et al., 2009; Kemp et al., 2012). After being cleaved from Ang II by the enzyme aminopeptidase A (APA), Ang III primarily binds and activates AT_2_ receptors to induce the Ang III/AT_2_/NHE3 signaling (Kemp et al., 2012; Zhuo and Li, 2019). This hypothesis is further supported by a recent study that intrarenal infusion of an AT_2_ receptor agonist C21 compound induced the subcellular translocation of membrane NHE3 in the proximal tubules of WKY but not SHRs (Kemp et al., 2020). This NHE3 internalization or translocation was associated with significantly increased natriuretic and vasoprotective responses in WKY, suggesting that the Ang III/AT_2_/NHE3 signaling pathway may play a counteract regulatory role in the regulation of proximal tubule Na^+^ reabsorption. The angiotensin-converting enzyme 2 (ACE2)/Ang-(1–7)/Mas receptor axis may also regulate NHE3 expression and activity in the kidney of SHRs (Castelo-Branco et al., 2017). Ang (1–7) is derived from Ang I or Ang II by the action of ACE2 and binds to the Mas receptor (Chappell, 2012; Karnik et al., 2017). Ang (1–7) elicits an antagonistic and protective response to Ang II primarily by promoting NO production and inducing vasodilation. Administration of high doses of Ang 1–7 has been shown to inhibit NHE3 transporter activity and decrease kidney Na^+^ reabsorption in SHRs (Castelo-Branco et al., 2017). Nevertheless, the roles of these alternative pathways in the regulation of NHE3 expression and activity and subsequent Na^+^ reabsorption from the proximal tubules, blood pressure control, and the development of Ang II-dependent hypertension may be much smaller than those of the predominant Ang II/AT_1_ (AT_1a_)/NHE3 signaling pathways.

## Sex Differences, NHE3 Expression, and Hypertension

Sex differences occur in every genetic, genomic, biological, physiological, and diseased responses, as expected. Indeed, it is well-established that the prevalence of hypertension varies between the sexes and increases with age (Silva-Antonialli et al., 2004; Choi et al., 2017; Ramirez and Sullivan, 2018). Males generally have a higher prevalence than premenopausal females, but this changes after menopause, with prevalence increasing in postmenopausal females (Choi et al., 2017; Ramirez and Sullivan, 2018). *In vivo* studies using SHRs have demonstrated sex differences in the RAS showing that female rats showed lower AT_1_ receptor expression and higher AT_2_ receptor expression in the kidney and vasculatures when compared to male SHRs (Silva-Antonialli et al., 2004). Hilliard et al. (2011, 2012) have likewise shown gender differences in AT_2_ receptor-mediated pressure-natriuresis and renal autoregulation. Additionally, Ang 1–7 was found to be increased in female SHRs despite no difference being found in Ang II levels between the sexes (Sullivan et al., 2010). We have recently studied sex differences in the hypertensive response to Ang II infusions, finding no differences in hypertensive responses between the male and female WT mice, but noting a significant decrease in hypertensive response to Ang II infusion between male and female PT-*Nhe3^−/−^* mice (Li et al., 2019b). In female PT-*Nhe3^−/−^* mice, the hypertensive response to chronic Ang II infusion was attenuated after 3 days, while in males the same response persisted for 7 days (Li et al., 2019b). Veiras et al. (2017) determined that in female rat proximal tubules, phosphorylation of NHE3 was markedly increased, but the expression of other sodium transporters further down the nephron segments was increased, resulting in a similar natriuresis response due to compensation. The mechanisms behind some but not all sex differences remain unclear, but female hormones, along with other unknown modifiers, may play a protective role in the development of hypertension (Seely et al., 1999; Mirabito et al., 2014; Hu et al., 2021).

## Perspectives on Therapeutically Targeting Intestinal and Kidney NHE3 in Hypertension

As reviewed and discussed above, the evidence presented in this review has highlighted the importance of NHE3 in the gastrointestinal tract, mainly small intestines, and the kidney, primarily the proximal tubules, in maintaining physiological Na^+^ and fluid balance, basal blood pressure homeostasis, the pressure-natriuresis response, and its role in the development of Ang II-induced hypertension. By studying the roles and the mechanisms by which NHE3 regulates Na^+^ absorption in the gut and Na^+^ reabsorption in the proximal tubules, which contributes not only to maintain normal blood pressure but also to the development of Ang II-induced hypertension, a translational relevance may be discovered to pharmacologically target NHE3 in hypertension. With currently available antihypertensive drugs, appropriately 50% of hypertensive patients have their blood pressure adequately controlled. However, some patients fail to control their hypertension even treated simultaneously with three to four different antihypertensive drugs and develop so-called resistant hypertension or apparent treatment resistant hypertension (aTRH; Calhoun et al., 2008; Carey, 2013, 2019; Whelton et al., 2017; Muntner et al., 2018). The prevalence of resistant hypertension or aTRH remains as high as 13%–30% in American adult population, implying that other mechanisms are at play.

Against this background, we have hypothesized that therapeutically targeting NHE3 may present us a new additional pathway to treat human hypertension. As proof-of-concept studies, gastrointestinal NHE3 inhibitors have been developed to treat several disease targets including hypertension, constipation, and hyperphosphatemia in elder patients (Linz et al., 2012, 2016, 2020; Li et al., 2019b; Kovesdy et al., 2021). One NHE3 blocker, SAR218034 (SAR), has been shown to increase fecal sodium excretion and decrease systolic blood pressure in lean old SHRs (Linz et al., 2012). Another NHE3 inhibitor derived from SAR218034, tenapanor, was tested in both rats and healthy human volunteers. This human clinical trial demonstrated an increase in stool Na^+^, indicating that the drug was working to block the absorption of Na^+^ from the gut effectively (Spencer et al., 2014). Both drugs, which are nonabsorbable after oral ingestion and only target NHE3 in intestinal apical membranes, exhibited minimally changed plasma concentrations, indicating minimal systemic effects and allowing other NHE proteins to function as they would physiologically (Linz et al., 2012; Spencer et al., 2014). Combinations of tenapanor and enalapril, an ACE inhibitor, yielded even more impressive results in nephrectomized rats, decreasing systolic blood pressure by more than 30 mmHg when co-administered (Spencer et al., 2014). These results suggest that nonabsorbable, intestine-targeting NHE3 inhibitors may be promising for the treatment of resistant hypertension in elder patients with constipation when combined with other already existing antihypertensive drugs by selectively inhibiting NHE3 and Na^+^ absorption from the gastrointestinal tract. However, tenapanor is reportedly contraindicated in young patients due to salt wasting and decreases in blood pressure (Spencer et al., 2014).

**Figure 6:**
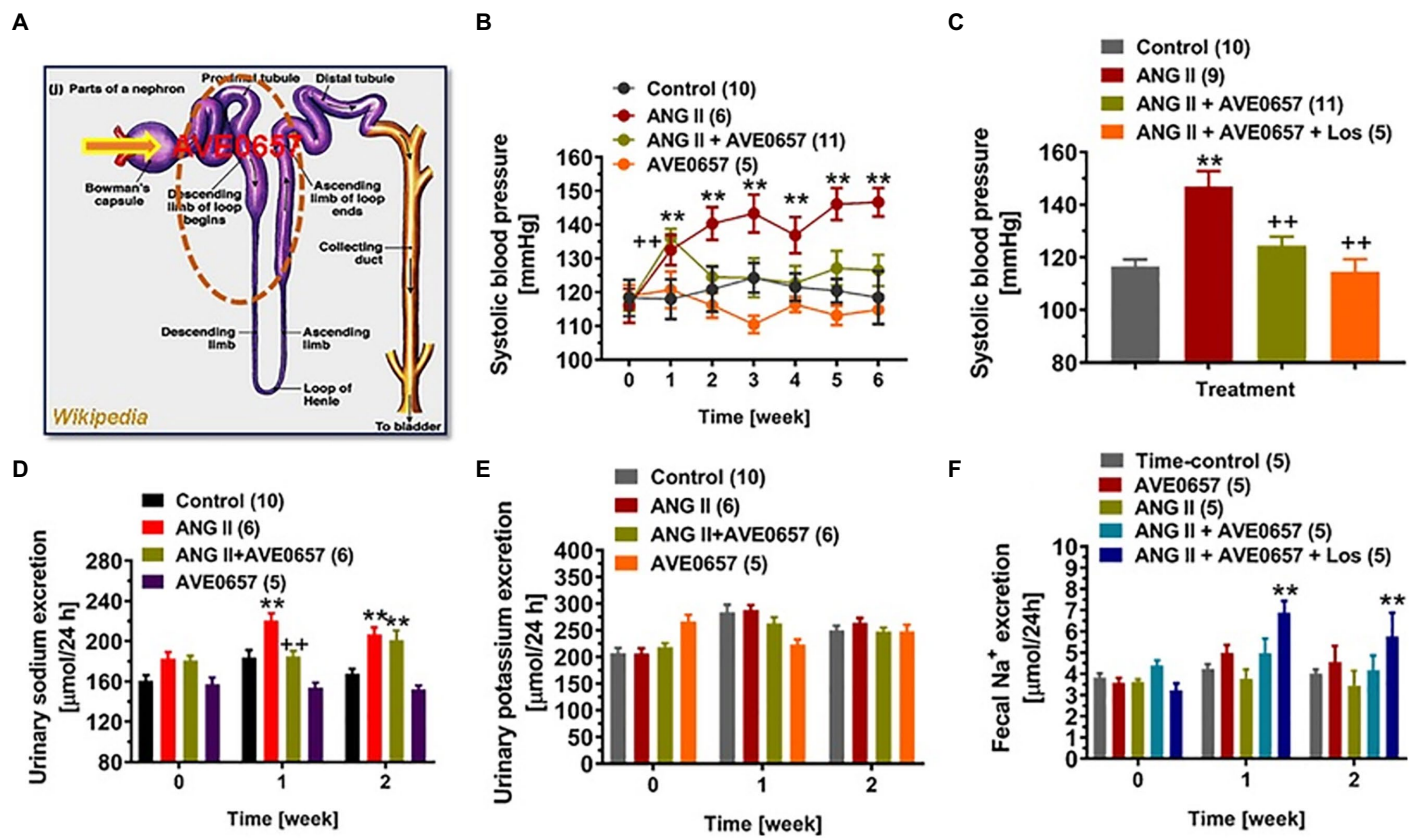
An orally absorbable NHE3 inhibitor AVE0657 (20 mg/kg/day, p.o., Sanofi-Aventis) significantly attenuated ANG II-induced hypertension in male C57BL/6 J mice infused with a slow pressor dose of ANG II at 0.5 mg/kg/day, i.p., for 2 weeks. Upon oral ingestion, AVE0657 is absorbable from the gut into the circulation, which is filtered by the glomerulus into the proximal tubules to inhibit NHE3 in the apical membranes **(A)**. **(B)** AVE0657 attenuated the development of Ang II-induced hypertension. **(C)** Concurrent treatments with AVE0657 and Ang II receptor blocker losartan completely blocked the development of Ang II-induced hypertension. **(D)** The effect of AVE0657 on natriuretic response. **(E)** AVE0657 had no effect on urinary potassium excretion. **(F)** AVE0657 had no effect on fecal sodium excretion. ^**^p < 0.01 vs. time-control group; ^++^*p* < 0.01 vs. ANG II group. Reproduced from Li et al. (2019b) with permission from the copyright holder.

A different class of NHE3 inhibitor, AVE-0657, has been developed by Sanofi-Aventis to treat sleep apnea (Wang et al., 2014), and related clinical trials have unfortunately abandoned. Unlike nonabsorbable SAR218034 and tenapanor (Linz et al., 2012; Spencer et al., 2014), AVE-0657 is absorbable from the gut after oral administration, enters the circulation, and is filtered by the glomerulus and expected to inhibit apical membrane NHE3 in the kidney, primarily the proximal tubules and less the thick ascending limb of the loop of Henle. We recently tested this hypothesis that AVE-0657 attenuates Ang II-induced hypertension in mice primarily by inhibiting NHE3 and Na^+^ reabsorption in the proximal tubules (Figure 6; Li et al., 2019b; Zhuo et al., 2021). Our studies demonstrated that AVE-0657 administration did not increase fecal Na^+^ excretion from the gastrointestinal tract suggesting that AVE-0657 does not inhibit NHE3 in small intestines as SAR218034 and tenapanor do. More importantly, AVE-0657 induced natriuresis and significantly attenuated the hypertensive response in Ang II-infused, 2% high salt-fed C57BL/6 J mice (Figure 6; Li et al., 2019b; Zhuo et al., 2021). When the ARB losartan was added to the AVE-0657 treatment, Ang II-infused, high salt-fed-indued hypertension was normalized to the control level. Interesting, the results of this study used AVE-0657 to treat Ang II-infused, high salt-fed-indued hypertensive mice are similar to or consistent with PT-*Nhe3^−/−^* mice with or without Ang II-infused, high salt-fed-indued hypertension (Li et al., 2018, 2019b). Taken together, these results are very promising, and we propose to further study and confirm this proof-of-concept hypothesis in different animal models of hypertension, especially in aTRH humans, by therapeutically targeting NHE3 in the proximal tubules of the kidney with or without other classes of antihypertensive drugs (Li et al., 2018, 2019b; Zhuo et al., 2021).

## Author Contributions

JZ and XL: conceptualization, review, and editing. SN: writing draft preparations. AL and RH: participants in experiments. SN, JZ, and XL: finalization. All authors contributed to the article and approved the submitted version.

## Funding

This work was supported in part by grants from National Institute of Diabetes and Digestive and Kidney Diseases (2R01DK102429-03A1, 2R01DK067299-10A1, and 1R01DK102429-01) and National Heart, Lung, and Blood Institute (1R56HL130988-01) to JZ.

## Conflict of Interest

The authors declare that the research was conducted in the absence of any commercial or financial relationships that could be construed as a potential conflict of interest.

## Publisher’s Note

All claims expressed in this article are solely those of the authors and do not necessarily represent those of their affiliated organizations, or those of the publisher, the editors and the reviewers. Any product that may be evaluated in this article, or claim that may be made by its manufacturer, is not guaranteed or endorsed by the publisher.
